# Whole-genome sequencing in clinically diagnosed Charcot–Marie–Tooth disease undiagnosed by whole-exome sequencing

**DOI:** 10.1093/braincomms/fcad139

**Published:** 2023-04-28

**Authors:** Young-gon Kim, Hyemi Kwon, Jong-ho Park, Soo Hyun Nam, Changhee Ha, Sunghwan Shin, Won Young Heo, Hye Jin Kim, Ki Wha Chung, Ja-Hyun Jang, Jong-Won Kim, Byung-Ok Choi

**Affiliations:** Department of Laboratory Medicine and Genetics, Samsung Medical Center, Sungkyunkwan University School of Medicine, Seoul 06351, Republic of Korea; Department of Neurology, Samsung Medical Center, Sungkyunkwan University School of Medicine, Seoul 06351, Republic of Korea; Clinical Genomics Center, Samsung Medical Center, Seoul 06351, Republic of Korea; Cell and Gene Therapy Institute (CGTI), Samsung Medical Center, Seoul 06351, Republic of Korea; Department of Laboratory Medicine and Genetics, Samsung Medical Center, Sungkyunkwan University School of Medicine, Seoul 06351, Republic of Korea; Department of Laboratory Medicine and Genetics, Samsung Medical Center, Sungkyunkwan University School of Medicine, Seoul 06351, Republic of Korea; Department of Laboratory Medicine and Genetics, Samsung Medical Center, Sungkyunkwan University School of Medicine, Seoul 06351, Republic of Korea; Department of Neurology, Samsung Medical Center, Sungkyunkwan University School of Medicine, Seoul 06351, Republic of Korea; Department of Biological Sciences, Kongju National University, Gongju 32588, South Korea; Department of Laboratory Medicine and Genetics, Samsung Medical Center, Sungkyunkwan University School of Medicine, Seoul 06351, Republic of Korea; Department of Laboratory Medicine and Genetics, Samsung Medical Center, Sungkyunkwan University School of Medicine, Seoul 06351, Republic of Korea; Clinical Genomics Center, Samsung Medical Center, Seoul 06351, Republic of Korea; Department of Neurology, Samsung Medical Center, Sungkyunkwan University School of Medicine, Seoul 06351, Republic of Korea; Cell and Gene Therapy Institute (CGTI), Samsung Medical Center, Seoul 06351, Republic of Korea; Samsung Advanced Institute for Health Sciences & Technology (SAIHST), Seoul 06351, Republic of Korea

**Keywords:** Charcot–Marie–Tooth disease, whole-genome sequencing, genotype-driven analysis, whole-exome sequencing

## Abstract

Whole-genome sequencing is the most comprehensive form of next-generation sequencing method. We aimed to assess the additional diagnostic yield of whole-genome sequencing in patients with clinically diagnosed Charcot–Marie–Tooth disease when compared with whole-exome sequencing, which has not been reported in the literature. Whole-genome sequencing was performed on 72 families whose genetic cause of clinically diagnosed Charcot–Marie–Tooth disease was not revealed after the whole-exome sequencing and 17p12 duplication screening. Among the included families, 14 (19.4%) acquired genetic diagnoses that were compatible with their phenotypes. The most common factor that led to the additional diagnosis in the whole-genome sequencing was genotype-driven analysis (four families, 4/14), in which a wider range of genes, not limited to peripheral neuropathy-related genes, were analysed. Another four families acquired diagnosis due to the inherent advantage of whole-genome sequencing such as better coverage than the whole-exome sequencing (two families, 2/14), structural variants (one family, 1/14) and non-coding variants (one family, 1/14). In conclusion, an evident gain in diagnostic yield was obtained from whole-genome sequencing of the whole-exome sequencing-negative cases. A wide range of genes, not limited to inherited peripheral neuropathy-related genes, should be targeted during whole-genome sequencing.

## Teaser

Kim *et al*. report the diagnostic yield of whole-genome sequencing from whole-exome sequencing-negative Charcot–Marie–Tooth disease patients. Among 72 families, 14 (19.4%) acquired genetic diagnosis by whole-genome sequencing. Whole-genome sequencing could be more widely used in the genetic diagnosis of Charcot–Marie–Tooth disease.

## Graphical Abstract

**Graphical abstract:**
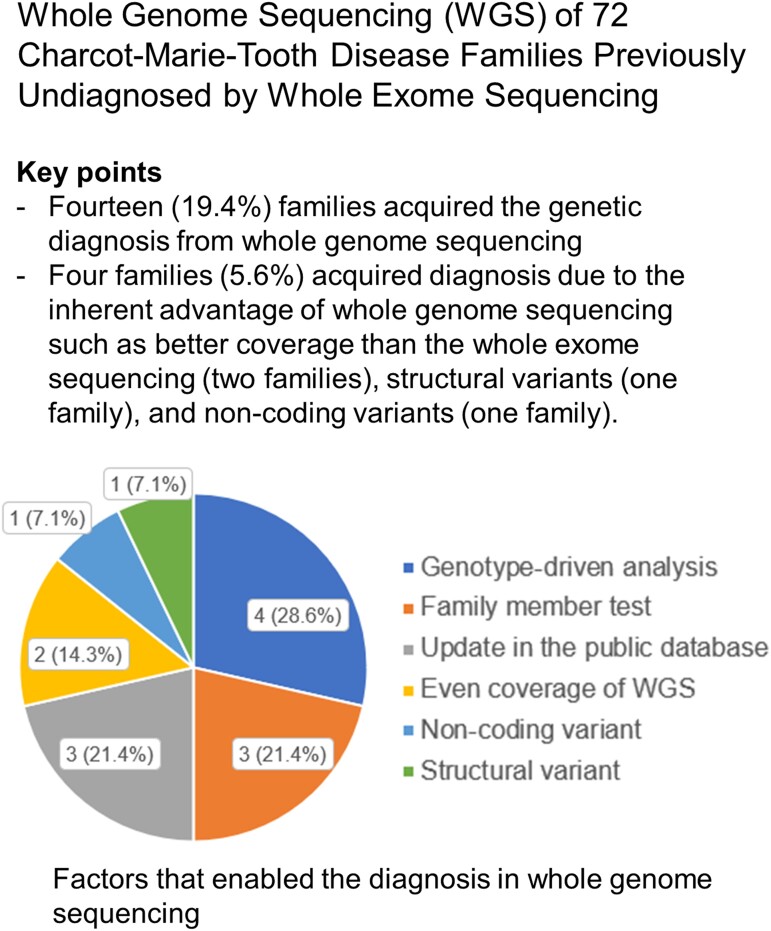

## Introduction

Charcot–Marie–Tooth disease (CMT) is a genetically and clinically heterogeneous group of disorders characterized by progressive distal limb weakness, gait disturbance and sensorimotor polyneuropathy.^1^ Owing to the advances in the field of genetic diagnosis such as next-generation sequencing, a large proportion of the CMT genetic landscape has been uncovered. This has led to the identification of more than 140 different causative genes for CMT.^1^ However, <60% of patients who are clinically diagnosed with CMT receive a genetic diagnosis.^2,3^

According to the target range, next-generation sequencing can generally be classified into three types of applications: targeted panel sequencing, whole-exome sequencing (WES) and whole-genome sequencing (WGS). In targeted panel sequencing, hundreds of genes associated with CMT are targeted and sequenced simultaneously to search for pathogenic variants. However, phenotypic overlaps between CMT and other inherited neuromuscular diseases such as hereditary spastic paraplegia, ataxia and distal myopathies are being increasingly recognized.^3–7^ In addition, the neuropathy observed in a patient with presumed CMT may be part of a more complex neurological or multisystem disorder. In this sense, WES can provide an additional diagnostic yield as the coding regions of all human genes can be assessed. WES can be performed in targeted panel sequencing-negative patients or as a primary test for CMT.^3,5–10^

WGS is the most comprehensive next-generation sequencing analysis because it can evaluate the entire range of the human genome, including non-coding regions. When compared with WES, the additional diagnostic yield of WGS can be attributed to its ability to read non-coding regions of the genome, such as deep-intronic regions, and increase the capability of detecting structural variants. Even for coding regions, it has been repeatedly reported that WGS shows better variant detection performance than WES due to better coverage, especially in GC-rich regions, owing to the lack of amplification steps during library preparation.^1,11–14^ It was reported that even within annotated coding exons, WES misses 10.7% of variants that were found through WGS.^14^

There are reports in which the genetic basis of inherited peripheral neuropathy including CMT could only be found by WGS. Brewer *et al*.^15^ identified a 78 kb insertion on chromosome 8 in the WGS of a family where no pathogenic variant was found with WES. In a study by Drew *et al*.,^16^ a 1.35 Mb insertion in the distal hereditary motor neuropathy Type 1 locus on chromosome 7q34-q36.2 was identified in a case where all other diagnostic procedures such as targeted panel sequencing, WES and chromosomal microarray failed to identify the genetic cause of inherited peripheral neuropathy.

In this study, we aimed to assess the diagnostic yield of WGS in CMT compared to that of WES. WGS was conducted in 72 families with clinically diagnosed CMT where no genetic diagnosis was obtained from WES and 17p12 duplication screening. The positive cases were further reviewed to determine the specific aspects of WGS that contributed to an additional diagnosis when compared to WES.

## Materials and methods

This study was performed under the National Project of Bio Big Data in South Korea and was approved by the Institutional Review Board of the Samsung Medical Center, Seoul, South Korea (IRB No.: SMC 2020–10-042).

### Participants

Informed consent for genetic testing was obtained from all included participants. Patients who were confirmed to have CMT by comprehensive examinations, including neurological examination, nerve conduction study and lower extremity MRI were eligible. Patients who were genetically undiagnosed after WES were selected at random for WGS, which was conducted from July 2020 to July 2021.

### Whole-exome sequencing and 17p12 duplication screening

The results of the WES and 17p12 duplication screening were reviewed for their inclusion. WES was performed on genomic DNA at Macrogen Inc. (Seoul, Korea) using the Human SeqCap EZ Human Exome Library v3.0 (Roche/NimbleGen, Madison, WI, USA) or the SureSelect Human All Exon Kit (Agilent Technologies, Santa Clara, CA, USA) as previously described.^17,18^ The 17p12 duplication screening was performed using hexaplex microsatellite PCR as described in a previous study.^19^

While all included probands had prior WES results, the raw data were not available for most of the cases because the expiration date of the sequencing service provider had passed. For cases in which quality control reports were available, the mean depth of coverage and 10 × coverage on the matched target region were compared between WES and WGS.

### Whole-genome sequencing

One microgram of genomic DNA was extracted from whole blood specimens. A WGS library was constructed using a TruSeq DNA PCR-Free Library Preparation Kit (Illumina, Inc., San Diego, CA, USA) in accordance with the manufacturer’s instructions. WGS was performed using the NovaSeq 6000 platform with paired-end reads of 150 bp, in accordance with the manufacturer’s instructions. The sequence reads were aligned to a human reference genome (hg38) using Burrows–Wheeler Alignment software, version 0.7.17.^20^ Data preprocessing and variant detection were performed using HaplotypeCaller of Genome Analysis Toolkit, version 4.2.0.^21^ Variant annotation was performed using ANNOVAR.^22^

For the interpretation of variants, both genotype-driven and phenotype-driven analyses were used as described in a recent article regarding best practices of WGS interpretation.^23^ In the phenotype-driven analysis, 287 genes associated with peripheral neuropathy were evaluated (Supplementary Table 8).

### Biochemical tests

For patients suspected of having Krabbe disease based on their pathogenic variants from *GALC*, galactocerebrosidase activity was measured as an outsourced test to the Mayo Clinic Laboratory. Leucocyte galactocerebrosidase activity was measured using liquid chromatography-tandem mass spectrometry, as described in the official website of the Mayo Clinic Laboratories (https://www.mayocliniclabs.com/test-catalog/Overview/606270#Performance, Accessed 2 November 2022).

For patients suspected to have sorbitol dehydrogenase deficiency due to their bi-allelic inactivating variants from *SORD*, fasting serum sorbitol levels were measured. Separated serum was sent to the Laboratory of IRCCS Mondino Foundation, Pavia, Italy, where sorbitol levels were measured in the discovery study. The sorbitol levels were measured using ultra-performance liquid chromatography-tandem mass spectrometry as described in the discovery study.^24^

### Statistical analysis

R (version 4.0.3) software was used for the statistical analysis. The Shapiro test was used to test the normality of the data. For the comparison of the two groups, the *t*-test was used when the normality assumption was satisfied; otherwise, the Mann–Whitney test was used.

## Results

### Patient information

**Figure 1:**
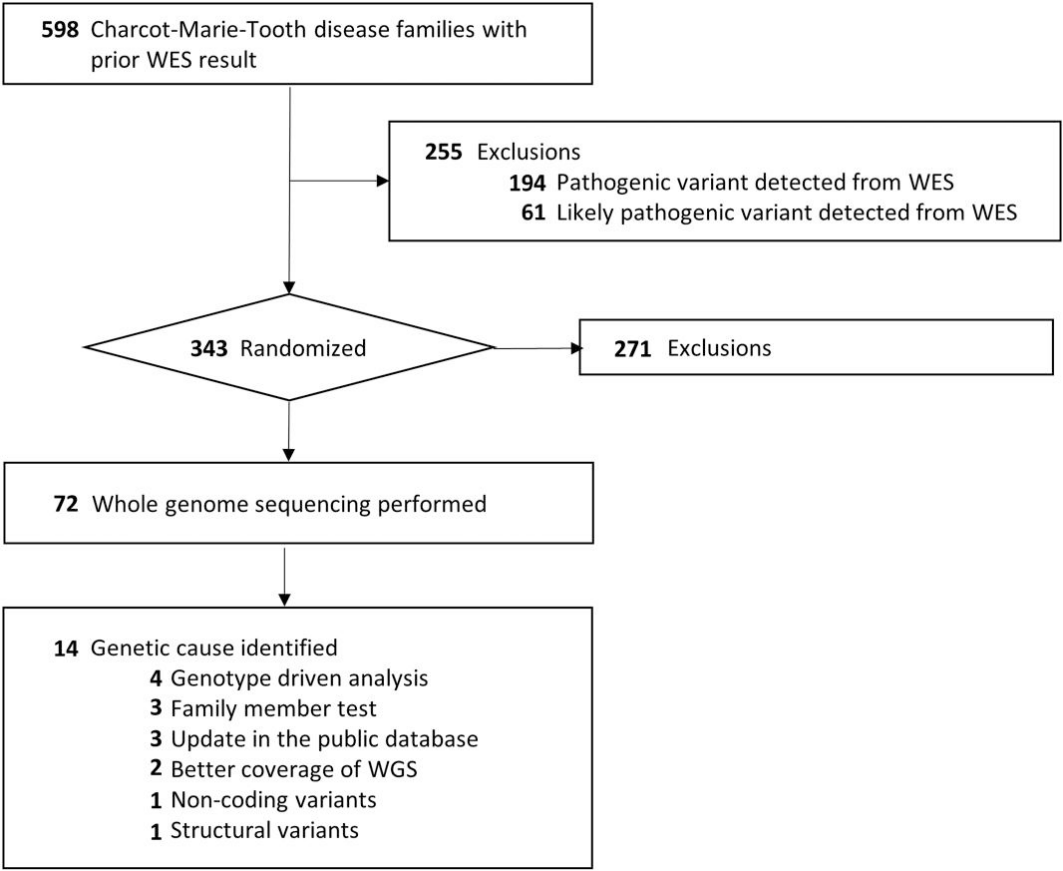
**Flowchart of study inclusion criteria and analysis procedure.** A total of 598 families visiting the Samsung Medical Center outpatient clinic for their peripheral neuropathy had been tested with WES. Among 343 families that remained genetically undiagnosed, 72 families selected randomly were included in the study. For the 14 families whose genetic cause was identified, factors that enabled the diagnosis were reviewed.

A total of 762 individuals from 598 families visiting the Samsung Medical Center outpatient clinic for CMT were tested using WES (Fig. 1). WES was performed from 2011 to 2021, and the study details of 56 WES papers are listed in Supplementary Table 1. Among the 343 families that remained genetically undiagnosed after WES, 72 families selected at random were included in this study.

A list of all the included cases is provided in Supplementary Table 2. Altogether, 72 patients with CMT satisfied the enrolment criteria, and 147 individuals, including family members, were subjected to WGS analysis. Among the 72 families, 32 families (44.4%) were trios. Among the trio families, 31 consisted of a proband and both parents and one family consisted of a proband, mother and affected brother. Eleven families were duos (15.3%), consisting of probands and either the mother (9/11) or the father (2/11). In addition, 29 families (40.3%) consisted of only probands. Five individuals who enrolled as family members also had CMT. Thus, in total, 77 patients with CMT were enrolled in this study.

### Whole-genome sequencing

For 147 individuals, including patients and unaffected family members, an average of 117.01 Gb of sequence was generated (Supplementary Table 3). The mean depth of coverage ranged from 26.4 to 62.6 (average: 35.3). The average 10 × coverage was 94.7% (range: 90.6–95.5%). A WES quality control report was available for 54 patients, and the depth of coverage was compared between WES and WGS for these cases (Supplementary Table 4). Because the patients were tested with WES over a wide time range, three different WES library kits were used. When the matched region was compared between WES and WGS, the 10 × coverage did not show a significant difference (WES 96.1%, WGS 96.5%, *P* = 0.333) despite the marked difference in the mean depth of coverage (WES 92.0, WGS 36.7, *P* < 0.001).

The genetic, phenotypic and electrophysiological findings of cases additionally diagnosed by WGS are shown in Table 1 and Supplementary Tables 5 and 6, respectively. Fourteen of the 72 families (19.4%) acquired phenotypically relevant genetic diagnoses from WGS. The mutated genes found in this study are *ABCD1, ATP7B, GALC, INF2, KIF1A, NEFL, PMP2, SACS, SLC40A1, SORD* and *SPTLC1*.

**Table 1:** Genetic findings of the additionally diagnosed cases from WGS

Family ID	Age/sex	Gene	OMIM phenotype (inheritance)	DNA change	Protein change	Observed inheritance	Factors enabled diagnosis in WGS
FAM03	38/M	SPTLC1	Neuropathy, hereditary sensory and autonomic, Type IA (AD)	c.992C>T	p.Ser331Phe	*De novo*	Better coverage of WGS ^a^Family member test
FAM10	13/M	PMP2	CMT 1G (AD)	c.47A>T	p.Asn16Ile	*De novo*	Family member test
FAM25	15/F	INF2	CMT, DI E (AD)	c.311G>A	p.Cys104Tyr	Unknown	Better coverage of WGS
FAM26	18/M, 20/M	KIF1A	Spastic paraplegia 30 (AD)	c.2022+1G>C	p.?	Unknown	Family member test
FAM30	25/M	SORD	Sorbitol dehydrogenase deficiency with peripheral neuropathy (AR)	c.[757del];[908+1G>A]	p.[Ala253GlnfsTer27];[?]	Paternal/not paternal (From additional father test)	Public database update
FAM31	25/M	ABCD1	Adrenomyeloneuropathy (XR)	c.593C>G	p.Thr198Arg	*De novo*	Genotype-driven analysis ^a^Family member test
FAM32	20/M	GALC	Krabbe disease (AR)	c.[857G>A];[Exon 17 deletion]	p.[Gly286Asp];[Exon 17 deletion]	Paternal/maternal	Structural variant
FAM33	38/M	ATP7B	Wilson disease (AR)	c.[2333G>T];[936G>C]	p.[Arg778Leu];[Gln312His]	Maternal/not maternal	Genotype-driven analysis
FAM37	22/M	SACS	Spastic ataxia, Charlevoix-Saguenay type (AR)	c.[3159_3160del];[1596T>A]	p.[Phe1054Ter];[Tyr532Ter]	Paternal/maternal	Genotype-driven analysis
FAM48	41/M	SLC40A1	Haemochromatosis, Type 4 (AD)	c.626>T	p.Ser209Leu	Paternal	Genotype-driven analysis
FAM59	32/F	GALC	Krabbe disease (AR)	c.[952C>G];[1901T>C]	p.[Pro318Ala][Leu634Ser]	Not maternal/maternal	Public database update
FAM61	19/F	KIF1A	NESCAV syndrome (AD)	c.76°C>T	p.Arg254Trp	*De novo*	Public database update
FAM63	49/F	NEFL	CMT, DI G (AD)	c.293A>G	p.Asn98Ser	*De novo*	Family member test
FAM70	31/M	SORD	Sorbitol dehydrogenase deficiency with peripheral neuropathy (AR)	c.[757del];[*2118T>G]	p.[Ala253GlnfsTer27];[?]	Maternal/paternal	Non-coding variant ^a^Public database update

a Secondary factor.

AD, autosomal dominant; AR, autosomal recessive; CMT, Charcot–Marie–Tooth disease; DI, dominant intermediate; F, female; M, mal; OMIM, online Mendelian inheritance in man.

### Factors that enabled an additional diagnosis in WGS compared to prior WES

**Figure 2:**
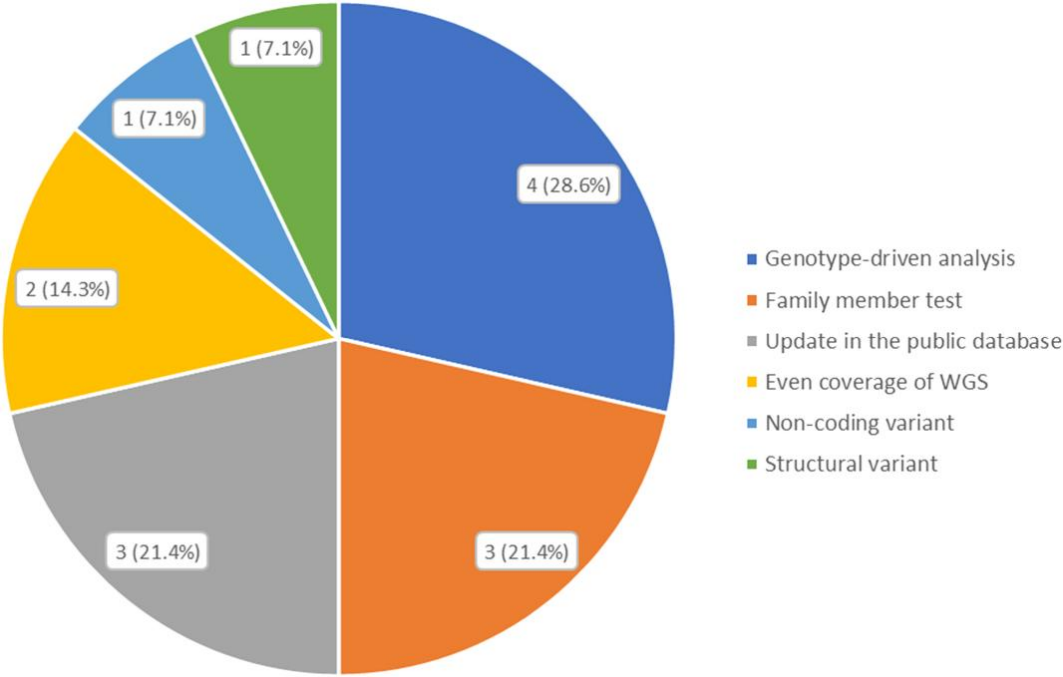
**Factors that enabled additional diagnosis using WGS (*n* = 14).** Genotype-driven analysis was the most common factor that enabled additional diagnosis in WGS (4/14, 28.6%). Other causes included family member test (3/14, 21.4%), update in the public database (3/14, 21.4%), better coverage of WGS (2/14, 14.3%), non-coding variant (1/14, 7.1%) and structural variant (1/14, 7.1%).

The factors that enabled an additional diagnosis in WGS are described in Table 1 and Fig. 2. The most common factor was the genotype-driven analysis (4/14, 28.6%). In these cases, a sole focus on the CMT-related genes in prior WES analysis led to detection failure. In the genotype-driven analysis performed in this study, variants were detected in genes related to more complex disorders that affect both the CNS and peripheral nervous system, or genes related to metabolic diseases, such as *ABCD1*, *ATP7B*, *SACS* and *SLC40A1*. In three cases (21.4%), the family member test enabled the diagnosis of WGS. In these cases, the *de novo* status or co-segregation of phenotypes revealed by family member tests, such as the trio test, enabled pathogenic classification of the variants. Another important factor was the update of the public database (3/14, 21.4%). In these cases, updated information from the public databases of disease–gene relationships or disease–variant relationships, such as ClinVar (http://www.clinvar.com/), which were not available at the time of WES, enabled the diagnosis. Other factors included better coverage by WGS (2/14, 14.3%), non-coding variants (1/14, 7.1%) and structural variants (1/14, 7.1%).

### Cases detected from genotype-driven analysis

FAM31-1 was clinically diagnosed as CMT due to sensorimotor polyneuropathy with progressive gait disturbance. *ABCD1* likely pathogenic variant was detected using WGS, suggesting a diagnosis of adrenomyeloneuropathy, and plasma very long chain fatty acid test confirmed the diagnosis (Supplementary Table 5). The inheritance pattern of *ABCD1* is X-linked, but the variant was absent not only from the father of the individual but also from the mother. This indicated a *de novo* occurrence. The pathogenic classification might not have been possible without the identification of the *de novo* status from the trio test. Consequently, another factor, in addition to the genotype-driven analysis, that may have enabled the diagnosis in the present case was family member testing.

FAM33-1 showed sensorimotor polyneuropathy, gait disturbance and limb muscle atrophy without the brain or eye manifestations of Wilson’s disease. Two *ATP7B* variants were detected using genotype-driven analysis in WGS, and subsequent serum copper and ceruloplasmin tests confirmed the diagnosis of Wilson’s disease.

FAM37-1 was clinically diagnosed as CMT due to the presence of sensorimotor polyneuropathy and gait ataxia. Brain MRI was performed after two pathogenic *SACS* variants were detected by WGS, and prominent cerebellar atrophy was revealed. *SACS* mutations are cause of autosomal recessive Spastic ataxia, Charlevoix-Saguenay type.

FAM48-1 had sensorimotor polyneuropathy accompanied by fatty changes in the muscles of the lower extremity. The *HFE4* pathogenic variant (a known cause of Type 4 hemochromatosis), which was not detected in the former WES, was identified from WGS, and the subsequent serum ferritin level (430 ng/ml) confirmed the diagnosis of Type 4 haemochromatosis. Impaired glucose tolerance is one of the manifestations of Type 4 haemochromatosis. In the present case, the patient had poorly controlled Type 2 diabetes mellitus, thereby explaining his neuropathy as diabetic peripheral neuropathy.

### Cases detected by better coverage of WGS

In two cases (14.3%), FAM03 and FAM25, the variants that were detected in WGS were absent in the annotation files of the WES tests. The 10 × coverage of WES performed in these two cases was 90.7% and 91.1%, respectively. A pathogenic variant of *SPTLC1*, which is a cause of hereditary sensory and autonomic neuropathy Type IA, was detected from FAM03-1. FAM03-1 had phenotypes very close to those reported in a previous report,^25^ showing sensorimotor polyneuropathy, hoarseness and respiratory difficulties. The patient also underwent cataract surgeries on both eyes. One INF2 pathogenic variant, which is a cause of CMT, dominant intermediate E, was detected from FAM25-1. FAM25-1 showed phenotypes consistent with sensorimotor polyneuropathy with proteinuria caused by *INF2* mutations.

### Cases detected from structural variant analysis

**Figure 3:**
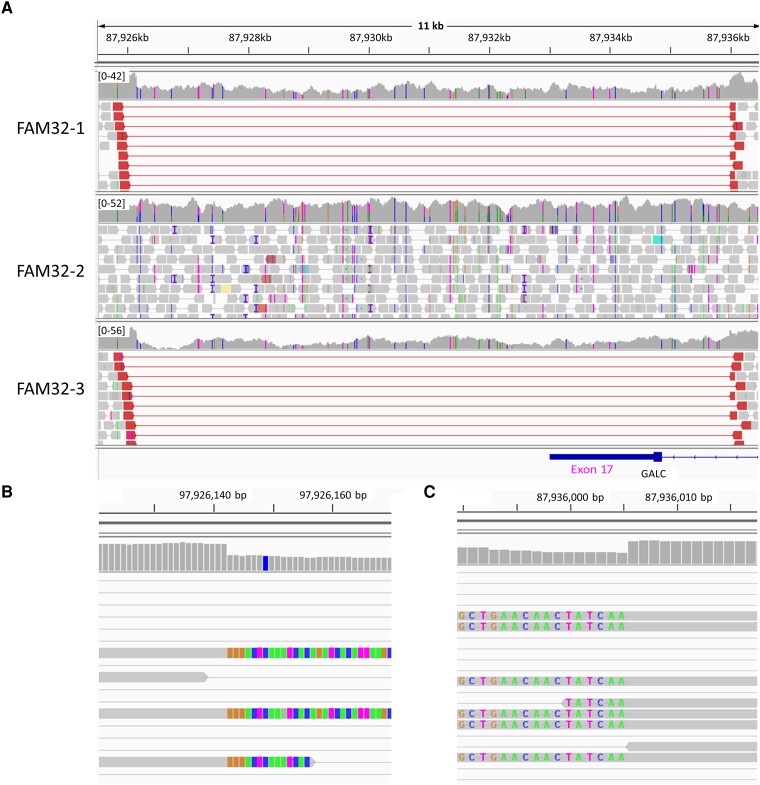
**BAM file findings of a large deletion encompassing *GALC* exon 17 detected in the FAM32 family**. (**A**) Discordantly paired reads and sudden decrease in the depth are observed from breakpoints. The deletion was detected from the proband (IPN32-1) and his mother (IPN32-3). Sudden decrease in the depth of coverage and soft-clipped reads are observed from the breakpoints. (**B**) 5′ breakpoint and (**C**) 3′ breakpoint.

FAM32-1 had sensorimotor polyneuropathy, distal limb muscle weakness and ataxia in both upper and lower limbs. He also had experienced seizures for 3 years and a brain MRI showed findings that were consistent with Krabbe disease. However, the diagnosis was not made until the two variants of *GALC* were found in WGS, and decreased galactocerebrosidase was subsequently identified. One of the two *GALC* variants had a large deletion, including exon 17, which could be definitively found in WGS (Fig. 3).

### Cases of SORD deficiency with peripheral neuropathy

**Figure 4:**
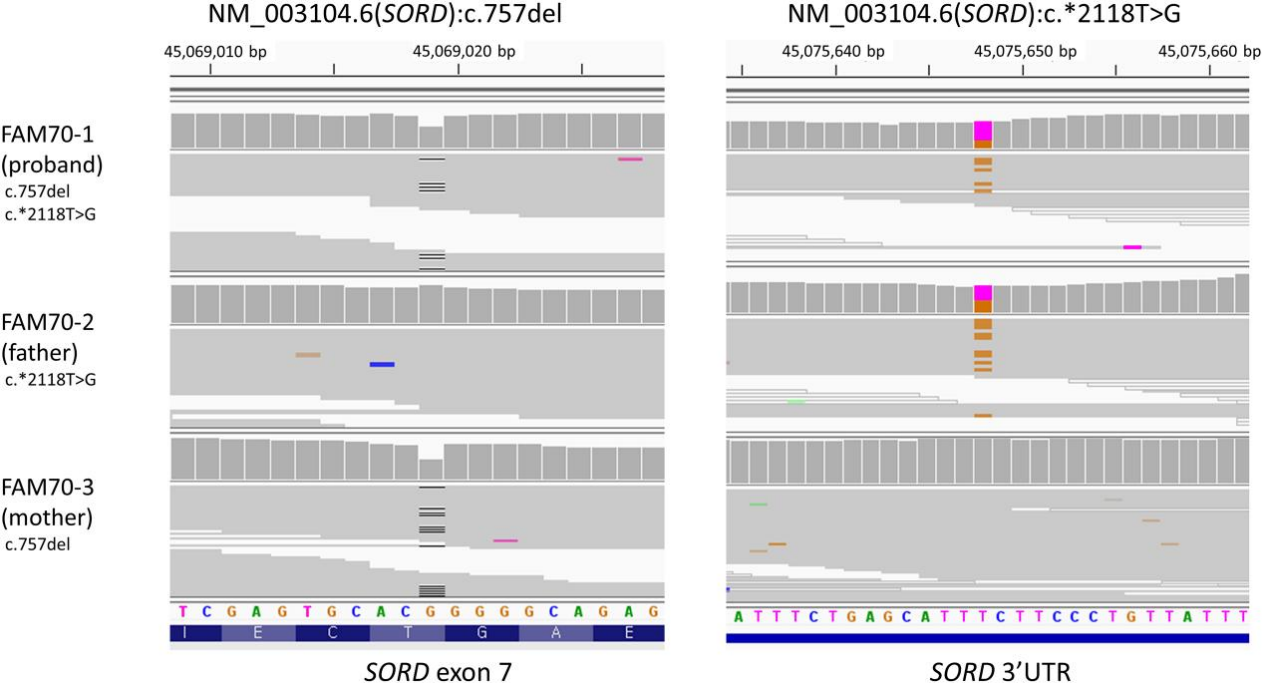
**BAM file findings of *SORD* variants in the FAM70 family.** The c.757del variant was detected in the proband and his mother. The 3′UTR variant, c.*2118T>G was detected in the proband and his father.

Both FAM30-1 and FAM70-1 had phenotypes consistent with CMT and failed to have a genetic diagnosis based on WES. In WGS, variants were detected in *SORD* (sorbitol dehydrogenase), which was recently identified as a causative gene of CMT or distal hereditary motor neuropathy (Fig. 4, Supplementary Table 7).^24,26,27^ In *SORD*, the c.757del variant is widely known as the most common pathogenic variant, and the majority of the cases reported so far are homozygous for this variant. In FAM30-1, one heterozygous c.757del variant and another heterozygous likely pathogenic variant, c.908+1G>A, were detected. The proband was enrolled for WGS in this family, but his father was additionally tested by Sanger sequencing, using primers published previously.^24^ He was confirmed to have c.757del and not c.908+1G>A, suggesting the trans-phasing of these two variants. Trio WGS was performed in the FAM70 family and compound heterozygous status in trans-phasing of the two variants, c.757del and c.*2118T>G, was confirmed. The fasting serum sorbitol level measured by the same method at the same laboratory in the discovery study^24^ confirmed the diagnosis of both families (Supplementary Table 7).

## Discussion

This is the first study to evaluate the diagnostic yield of WGS from WES-negative cases in the field of CMT. The additional diagnostic yield was 19.4%. The most common factor that enabled the diagnosis using WGS was the genotype-driven analysis. There are two approaches for prioritizing variants detected by WES or WGS: phenotype-driven analysis and genotype-driven analysis.^23^ In phenotype-driven analysis, a virtual panel of genes associated with a specific patient phenotype becomes the target for downstream analysis. However, in genotype-driven analysis, all potential pathogenic variants are considered regardless of the genes. The genotype-driven analysis used in this study contributed the most to the diagnostic yield, demonstrating that CMT has considerable phenotypic overlap with other categories of disorders. Except for six families (8.3% of 72) whose final genetic diagnosis was compatible with CMT (FAM03, FAM10, FAM25, FAM30, FAM63 and FAM70), the final diagnoses of the other eight families (11.1% of 72) were not typical CMT although clinical features including electrophysiological and neuroimaging findings were similar to CMT. Thus, targeting only CMT-related genes in patients with presumed CMT might result in the exclusion of a considerable proportion of genetic diagnoses.

Genotype-driven analysis can also be performed in WES and its importance has been demonstrated in previous studies.^5–7^ However, we assume that genotype-driven analysis of WGS might be more efficient than that of WES, because the impact of pathogenic variants from unexpected genes is much higher in WGS than in WES. In WES, for a variant detected from a gene with an indefinite disease–gene relationship, another undetected genetic cause could be assumed, probably in a non-coding region or poorly covered region, hampering the plausibility of the detected variant. However, if the same variant was detected from WGS and the variant was considered the most probable, a much higher priority could be given because the variant would be considered most probable from the entire genome. We believe this can at least partly explain why the variants detected by genotype-driven analysis in this study were not detected in the prior WES.

The four cases (5.6% of 72) that were diagnosed due to the better coverage of WGS, structural variants and non-coding variants, could not be diagnosed by WES due to its inherent limitations. In this sense, the purely additional yield of WGS compared to WES in the current study may be considered as 5.6%. Considering WES missed 10.7% of coding variants and 12.5% of splicing variants that were detected by WGS in a recent report (from UK Biobank),^14^ this proportion was smaller than expected, although these two measures, the diagnostic yield and the proportion of variants missed by WES, are not directly comparable.

We assume that this is due to the limitation of our study, the lack of matched raw WES data in most cases. The annotation files and quality control reports were available from a limited number of cases with CMT, and most of the variants detected from WGS could not be reviewed from the WES raw data. Otherwise, if the variants detected from WGS were not detected from WES raw data, there would have been more variants confirmed to be detected only in WGS due to the better coverage. In a study that compared the coverage performance of WES and WGS systematically, 13× coverage was better in WGS from exons of all RefSeq genes, especially from the first exon of genes.^12^ This is in contrast to the misunderstanding that for coding regions, WES has higher coverage than WGS. A future study incorporating the complete set of raw data from both WES and WGS could derive the true advantage of WGS in terms of coverage.

Non-coding variants can never be detected with other testing methods such as WES. The variants in 3′ untranslated region detected from FAM70 were the only non-coding variant detected in this study. We assume that this is due to the prematurity of interpretation techniques and guidelines for non-coding variants. Since the non-coding variant interpretation is one of the most active areas of research, the accessibility to non-coding regions by WGS will be appreciated more highly in the future.

In conclusion, this was the first study that evaluated the additional diagnostic yield of WGS after performing WES in patients with clinically diagnosed CMT. Fourteen families (14/72, 19.4%) who failed to acquire a genetic diagnosis from WES were diagnosed using WGS. A wider range of genes should be targeted in WGS using genotype-driven analysis. In addition to the well-known advantages of WGS compared with WES, such as structural variants and non-coding variants, better coverage even in the coding regions could be another important advantage of WGS.

## Supplementary Material

fcad139_Supplementary_Data

## Data Availability

All raw data used and/or analysed during the current study are available from the corresponding author upon request.

## Abbreviations

CMT =: Charcot–Marie–Tooth disease
SMC =: Samsung Medical Center
WES =: whole-exome sequencing
WGS =: whole-genome sequencing
