# Supplementary material for: Whole-genome sequencing in clinically diagnosed Charcot–Marie–Tooth disease undiagnosed by whole-exome sequencing
Source: Brain Commun. 2023 Apr 28;5(3):fcad139. doi: 10.1093/braincomms/fcad139 (PMC10174204; doi:10.1093/braincomms/fcad139)
Supplement: fcad139_Supplementary_Data [file fcad139_supplementary_data.zip › Supplementary Table 1 and Supplementary References.docx]

**Supplementary Table 1.** List of variants detected from previous WES studies carried out by the authors

| **Reference No.** | **Gene** | **Nucleotide substitution** | **Year** | **Journal** |
| --- | --- | --- | --- | --- |
| 1 | *GARS* | c.598G>A, c.794C>T | 2012 | J Peripher Nerv Syst. |
| 2 | *NEFL*  *MPZ*  *MFN2*  *PMP22*  *GDAP1* | c.1150A>T, c.1186G>A, c.64C>A  c.449-1G>T  c.1090C>T  c.325T>C, c.215C>T  c.677G>A | 2012 | Hum Mutat. |
| 3 | *Cx32* | c.13G>A, c.77delC, c.109G>T, c.257C>T, c.454delG, c.458T>G, c.536-537insACTG, c.590C>T, c. 641-642TC>AT | 2012 | Clin Genet. |
| 4 | *MFN2* | c.1048T>C, c.310C>T | 2012 | Genes Genom. |
| 5 | *TFG* | c.854CT | 2013 | JAMA Neurol. |
| 6 | *SBF1* | c.1249A>G + c.4768A>G | 2013 | Neurology |
| 7 | *PLEKHG5* | c.1988C>T, c.2458G>C | 2013 | Orphanet J Rare Dis. |
| 8 | *HSPB8* | c.422A>C | 2013 | Neuromuscul Disord. |
| 9 | *MYH7* | c.4315G>C | 2013 | Neuromuscul Disord. |
| 10 | *TYMP* | c.451A>C + c.809T>C | 2013 | Mol Med Rep. |
| 11 | *BSCL2* | c.269C>G | 2013 | Neurogenetics. |
| 12 | *PRPS1* | c.362C>G | 2013 | J Clin Neurol. |
| 13 | *MFN2* | c.839G>A, c.776G>A, c.292A>C | 2013 | Animal Cells and Systems |
| 14 | *HADHB* | c.210-1G > C + c.686G > T | 2013 | BMC Med Genet. |
| 15 | *INF2* | c.395T>C | 2014 | J Peripher Nerv Syst. |
| 16 | *MARS*  *YARS* | c.2398C>A  c.241_242GA>AT | 2014 | Clin Genet. |
| 17 | *SPTLC1* | c.992C>T | 2014 | Mol Med Rep. |
| 18 | *C10orf2* | c.1460C>T, c.1485-1G>A | 2014 | Neurogenetics. |
| 19 | *12SrRNA*  *tRNA-Ser*  *CO2* | m.1906T>C  m7471InsC  m.8108A>G | 2014 | J Genet |
| 20 | *MFN2* | c.275T>C, c.280C>T, c.292A>C, c.310C>T, c.314C>T, c.380G>A, c.494A>G, c.559A>G, c.617C>T, c.776G>C, c.787T>C, c.839G>A, c. 1048T>C, c.1085C>T, c.1090C>T, c.1127T>C, c.1253G>A, c.1367C>T, c.1525G>C, c.1930_1932delCTC, c.2230G>A | 2015 | Clin Genet. |
| 21 | *TUBB3* | c.1249G>A | 2015 | Mol Med Rep. |
| 22 | *MPV17* | c.122G> A | 2015 | BMC Neurol. |
| 23 | *COL6A1* | c.1056+1G>A | 2015 | J Clin Neurol. |
| 24 | *PRX* | c.1174C>T + c.2035C>T | 2015 | J Clin Neurol. |
| 25 | *FGD4* | c.1512-2A>C, c.2043+1G>A | 2015 | Ann Hum Genet. |
| 26 | *HSPB1* | c.404C[T | 2015 | Genes Genom. |
| 27 | *MORC2* | c.73C>T, c.209G>T, c.568C>T | 2016 | Brain |
| 28 | *ADSSL1* | c.910G>A, c.1048delA | 2016 | Ann Neurol. |
| 29 | *PDK3* | c.473G>A | 2016 | J Peripher Nerv Syst. |
| 30 | *DGAT2* | c.667T>C | 2016 | Hum Mutat. |
| 31 | *PMP22*  *SH3TC2*  *MPZ*  *GJB1*  *SPTLC2*  *DCTN1* | c.47T>C  c.929G > A+ c.3272G > T  c.154T>G, c.262T>C  c.286G>C  c.435G>T  c.1019A>G | 2016 | Mol Cells. |
| 32 | *DCTN1* | c.175G>A | 2016 | Mol Med Rep. |
| 33 | *OPA1* | c.1857‑1858delinsT, c.G2714A | 2016 | Mol Med Rep. |
| 34 | *PMP22* | c.245T>A, c.215C>T | 2016 | Genes Genom. |
| 35 | *GALC* | c.1687A>T + c.1901T>C  c.683_694delinsCTC + c.857G>A | 2016 | Oncotarget. |
| 36 | *GJB1* | c.3G>T, c.7T>C, c.13G>A, c.20A>G, c.22A>C, c.43C>T, c.44G>A, c.47A>G, c.62G>A, c.77C>T, c.77delC, c.109G>T, c.112G>T, c.137A>G, c.139G>A, c.157T>C, c.257C>T, c.271G>A, c.283G>A, c.286G>C, c.328G>A, c.394T>C, c.407T>C, c.414C>G, c.415G>A, c.424C>T | 2017 | J Peripher Nerv Syst. |
| 37 | *NEFH* | c.3015_3027dup | 2017 | J Peripher Nerv Syst. |
| 38 | *FASTKD2* | c.613T>C, c.764T>C | 2017 | Mitochondrion. |
| 39 | *PMP22* | c.325T>C, c215C>T | 2017 | Neurol India. |
| 40 | *HSPB3* | c.352T>C | 2018 | J Peripher Nerv Syst. |
| 41 | *LGI1* | c.988C>T | 2018 | J Clin Neurol. |
| 42 | *BAG3* | c.626C>T | 2018 | Genes Genom. |
| 43 | *KIF5A* | c.610C>T, c.839G>A, c.1673C>T | 2018 | Genes Genom. |
| 44 | *SH3TC2* | c.929G>A, c.2831A>G, c.929G>A, c.3272G>T | 2019 | J Clin Neurol. |
| 45 | *POLG2* | c.694G>A | 2019 | J Clin Neurol. |
| 46 | *EBP50* | c.973A>G | 2020 | GLIA |
| 47 | *AARS1* | c.2564A>G | 2020 | Genes Genom. |
| 48 | *SH3TC2*  *HK1*  *REEP1*  *MFN2* | c.2599C > T, c.3650G> A  c.19C > T  c.247delG  c.334G | 2021 | BMC Med Genomics. |
| 49 | *GNB4* | c.229G>A, c.265A>G | 2021 | Life (Basal) |
| 50 | *GDAP1* | c.332C>A, c.358C>T, c.652C>G, c.656T>G, c.767A>G | 2021 | J Clin Neurol. |
| 51 | *MPZ* | c.155T>G, c.358_360del, c.394C>T, c.398C>T, c.659A>G | 2021 | Mol Genet Genomic Med. |
| 52 | *GARS1*  *AARS1*  *HARS1*  *WARS1*  *YARS1* | c.598G>A, c.794C>T, c.1007C>A, c.2171C>A  c.986G>A, c.1168C>T  c.395C>T, c.1147A>G  c.751G>A, c.1067A>T  c.241_242delGAinsAT, c.497A>G | 2022 | J Peripher Nerv Syst. |
| 53 | *PMP22* | c.35A>G, c.47T>G, c.68C>G, c.179-1G>A, c.215C>T, c.245T>C, c.256C>T, c.281delG, c.280_281delinsT, c.298G>A, c.318delT, c.319-1G>T, c.323T>C, c.325T>C | 2022 | Genes (Basel). |
| 54 | *HSPB1*  *HSPB8*  *HSPB3* | c.380G>A, c.404C>T, c.424T>C, c.544C>T, c.560C>T  c.412A>G, c.422A>C, c.423G>T  c.352T>C | 2022 | Genes (Basel). |
| 55 | *MYH14* | c.2822G>T | 2022 | J Clin Neurol. |
| 56 | *NEFL* | c.935T>C, c.1327T>A, c.1401G>C | 2022 | Mol Genet Genomic Med. |
| 57 | *LITAF* | c.334G>A | 2022 | Genes Genom. |

**Supplementary references**

**1.** Lee HJ, Park J, Nakhro K, et al. Two novel mutations of GARS in Korean families with distal hereditary motor neuropathy type V*. J Peripher Nerv Syst*. 2012;17(4):418-421. doi:10.1111/j.1529-8027.2012.00442.x

**2.** Choi BO, Koo SK, Park MH, et al. Exome sequencing is an efficient tool for genetic screening of Charcot-Marie-Tooth disease*. Hum Mutat*. 2012;33(11):1610-1615. doi:10.1002/humu.22143

**3.** Kim Y, Choi KG, Park KD, Lee KS, Chung KW, Choi BO. X-linked dominant Charcot-Marie-Tooth disease with connexin 32 (Cx32) mutations in Koreans*. Clin Genet*. 2012;81(2):142-149. doi:10.1111/j.1399-0004.2011.01642.x

**4.** Nakhro K, Kim YJ, Lee JH, Koo H, Choi BO, Chung KW. Two de novo mutations of MFN2 associated with early-onset Charcot-Marie-Tooth disease type 2A neuropathy*. Genes Genom*. 2012;34:653–661.

**5.** Lee SS, Lee HJ, Park JM, et al. Proximal dominant hereditary motor and sensory neuropathy with proximal dominance association with mutation in the TRK-fused gene*. JAMA Neurol*. 2013;70(5):607-615. doi:10.1001/jamaneurol.2013.1250

**6.** Nakhro K, Park JM, Hong YB, et al. SET binding factor 1 (SBF1) mutation causes Charcot-Marie-Tooth disease type 4B3*. Neurology*. 2013;81(2):165-173. doi:10.1212/WNL.0b013e31829a3421

**7.** Kim HJ, Hong YB, Park JM, et al. Mutations in the PLEKHG5 gene is relevant with autosomal recessive intermediate Charcot-Marie-Tooth disease*. Orphanet J Rare Dis*. 2013;8:104. doi:10.1186/1750-1172-8-104

**8.** Nakhro K, Park JM, Kim YJ, et al. A novel Lys141Thr mutation in small heat shock protein 22 (HSPB8) gene in Charcot-Marie-Tooth disease type 2L*. Neuromuscul Disord*. 2013;23(8):656-663. doi:10.1016/j.nmd.2013.05.009

**9.** Park JM, Kim YJ, Yoo JH, et al. A novel MYH7 mutation with prominent paraspinal and proximal muscle involvement*. Neuromuscul Disord*. 2013;23(7):580-586. doi:10.1016/j.nmd.2013.04.003

**10.** Suh BC, Jeong HN, Yoon BS, et al. Compound heterozygous mutations of TYMP as underlying causes of mitochondrial neurogastrointestinal encephalomyopathy (MNGIE)*. Mol Med Rep*. 2013;8(1):17-22. doi:10.3892/mmr.2013.1479

**11.** Choi BO, Park MH, Chung KW, et al. Clinical and histopathological study of Charcot-Marie-Tooth neuropathy with a novel S90W mutation in BSCL2*. Neurogenetics*. 2013;14(1):35-42. doi:10.1007/s10048-012-0346-5

**12.** Park J, Hyun YS, Kim YJ, et al. Exome Sequencing Reveals a Novel PRPS1 Mutation in a Family with CMTX5 without Optic Atrophy*. J Clin Neurol*. 2013;9(4):283-288. doi:10.3988/jcn.2013.9.4.283

**13.** Nakhro K, Park JM, Choi BO, Chung KW. Missense mutations of mitofusin 2 in axonal Charcot–Marie–Tooth neuropathy: polymorphic or incomplete penetration? *Animal Cells Syst (Seoul)*. 2013;17(4):228-236.

**14.** Hong YB, Lee JH, Park JM, et al. A compound heterozygous mutation in HADHB gene causes an axonal Charcot-Marie-tooth disease*. BMC Med Genet*. 2013;14:125. doi:10.1186/1471-2350-14-125

**15.** Park HJ, Kim HJ, Hong YB, Nam SH, Chung KW, Choi BO. A novel INF2 mutation in a Korean family with autosomal dominant intermediate Charcot-Marie-Tooth disease and focal segmental glomerulosclerosis*. J Peripher Nerv Syst*. 2014;19(2):175-179. doi:10.1111/jns5.12062

**16.** Hyun YS, Park HJ, Heo SH, et al. Rare variants in methionyl- and tyrosyl-tRNA synthetase genes in late-onset autosomal dominant Charcot-Marie-Tooth neuropathy*. Clin Genet*. 2014;86(6):592-594. doi:10.1111/cge.12327

**17.** Suh BC, Hong YB, Nakhro K, Nam SH, Chung KW, Choi BO. Early-onset severe hereditary sensory and autonomic neuropathy type 1 with S331F SPTLC1 mutation*. Mol Med Rep*. 2014;9(2):481-486. doi:10.3892/mmr.2013.1808

**18.** Park MH, Woo HM, Hong YB, et al. Recessive C10orf2 mutations in a family with infantile-onset spinocerebellar ataxia, sensorimotor polyneuropathy, and myopathy*. Neurogenetics*. 2014;15(3):171-182. doi:10.1007/s10048-014-0405-1

**19.** Park JH, Yoon BR, Kim HJ, Lee PH, Choi BO, Chung KW. Compound mitochondrial DNA mutations in a neurological patient with ataxia, myoclonus and deafness*. J Genet*. 2014;93(1):173-177. doi:10.1007/s12041-014-0317-8

**20.** Choi BO, Nakhro K, Park HJ, et al. A cohort study of MFN2 mutations and phenotypic spectrums in Charcot-Marie-Tooth disease 2A patients*. Clin Genet*. 2015;87(6):594-598. doi:10.1111/cge.12432

**21.** Hong YB, Lee JH, Park HJ, et al. A family with axonal sensorimotor polyneuropathy with TUBB3 mutation*. Mol Med Rep*. 2015;11(4):2729-2734. doi:10.3892/mmr.2014.3047

**22.** Choi YR, Hong YB, Jung SC, et al. A novel homozygous MPV17 mutation in two families with axonal sensorimotor polyneuropathy*. BMC Neurol*. 2015;15:179. doi:10.1186/s12883-015-0430-1

**23.** Park HJ, Choi YC, Kim SM, et al. Molecular Genetic Diagnosis of a Bethlem Myopathy Family with an Autosomal-Dominant COL6A1 Mutation, as Evidenced by Exome Sequencing*. J Clin Neurol*. 2015;11(2):183-187. doi:10.3988/jcn.2015.11.2.183

**24.** Choi YJ, Hyun YS, Nam SH, et al. Novel Compound Heterozygous Nonsense PRX Mutations in a Korean Dejerine-Sottas Neuropathy Family*. J Clin Neurol*. 2015;11(1):92-96. doi:10.3988/jcn.2015.11.1.92

**25.** Hyun YS, Lee J, Kim HJ, et al. Charcot-Marie-Tooth Disease Type 4H Resulting from Compound Heterozygous Mutations in FGD4 from Nonconsanguineous Korean Families*. Ann Hum Genet*. 2015;79(6):460-469. doi:10.1111/ahg.12134

**26.** Kim HJ, Lee J, Hong YB, et al. Ser135Phe mutation in HSPB1 (HSP27) from Charcot–Marie–Tooth disease type 2F families*. Genes Genom*. 2015;37:295–303.

**27.** Hyun YS, Hong YB, Choi BO, Chung KW. Clinico-genetics in Korean Charcot-Marie-Tooth disease type 2Z with MORC2 mutations*. Brain*. 2016;139(Pt 7):e40. doi:10.1093/brain/aww082

**28.** Park HJ, Hong YB, Choi YC, et al. ADSSL1 mutation relevant to autosomal recessive adolescent onset distal myopathy*. Ann Neurol*. 2016;79(2):231-243. doi:10.1002/ana.24550

**29.** Kennerson ML, Kim EJ, Siddell A, et al. X-linked Charcot-Marie-Tooth disease type 6 (CMTX6) patients with a p.R158H mutation in the pyruvate dehydrogenase kinase isoenzyme 3 gene*. J Peripher Nerv Syst*. 2016;21(1):45-51. doi:10.1111/jns.12160

**30.** Hong YB, Kang J, Kim JH, et al. DGAT2 Mutation in a Family with Autosomal-Dominant Early-Onset Axonal Charcot-Marie-Tooth Disease*. Hum Mutat*. 2016;37(5):473-480. doi:10.1002/humu.22959

**31.** Nam SH, Hong YB, Hyun YS, et al. Identification of Genetic Causes of Inherited Peripheral Neuropathies by Targeted Gene Panel Sequencing*. Mol Cells*. 2016;39(5):382-388. doi:10.14348/molcells.2016.2288

**32.** Hwang SH, Kim EJ, Hong YB, et al. Distal hereditary motor neuropathy type 7B with Dynactin 1 mutation*. Mol Med Rep*. 2016;14(4):3362-3368. doi:10.3892/mmr.2016.5664

**33.** Lee J, Jung SC, Hong YB, et al. Recessive optic atrophy, sensorimotor neuropathy and cataract associated with novel compound heterozygous mutations in OPA1*. Mol Med Rep*. 2016;14(1):33-40. doi:10.3892/mmr.2016.5209

**34.** Kim JY, Koo H, Park KD, et al. Genotype–phenotype correlation of Charcot-Marie-Tooth type 1E patients with PMP22 mutations*. Genes Genom*. 2016;38:659–667.

**35.** Lim SM, Choi BO, Oh SI, et al. Patient fibroblasts-derived induced neurons demonstrate autonomous neuronal defects in adult-onset Krabbe disease*. Oncotarget*. 2016;7(46):74496-74509. doi:10.18632/oncotarget.12812

**36.** Hong YB, Park JM, Yu JS, et al. Clinical characterization and genetic analysis of Korean patients with X-linked Charcot-Marie-Tooth disease type 1*. J Peripher Nerv Syst*. 2017;22(3):172-181. doi:10.1111/jns.12217

**37.** Nam DE, Jung SC, Yoo DH, et al. Axonal Charcot-Marie-Tooth neuropathy concurrent with distal and proximal weakness by translational elongation of the 3' UTR in NEFH*. J Peripher Nerv Syst*. 2017;22(3):200-207. doi:10.1111/jns.12223

**38.** Yoo DH, Choi YC, Nam DE, et al. Identification of FASTKD2 compound heterozygous mutations as the underlying cause of autosomal recessive MELAS-like syndrome*. Mitochondrion*. 2017;35:54-58. doi:10.1016/j.mito.2017.05.005

**39.** Kim JY, Kim SH, Park JY, et al. A longitudinal clinicopathological study of two unrelated patients with Charcot-Marie-Tooth disease type 1E*. Neurol India*. 2017;65(4):893-895. doi:10.4103/neuroindia.NI_783_16

**40.** Nam DE, Nam SH, Lee AJ, Hong YB, Choi BO, Chung KW. Small heat shock protein B3 (HSPB3) mutation in an axonal Charcot-Marie-Tooth disease family*. J Peripher Nerv Syst*. 2018;23(1):60-66. doi:10.1111/jns.12249

**41.**  Kanwal S, Yoo DH, Tahir S, Lee SJ, Lee MH, Choi BO, Chung KW. A Novel Nonsense Mutation in Leucine-Rich, Glioma-Inactivated-1 Gene as the Underlying Cause of Familial Temporal Lobe Epilepsy. J Clin Neurol. 2018 Oct;14(4):591-593. doi:10.3988/jcn.2018.14.4.591.

**42.** Kim SJ, Nam SH, Kanwal S, et al. BAG3 mutation in a patient with atypical phenotypes of myofibrillar myopathy and Charcot-Marie-Tooth disease*. Genes Genomics*. 2018;40(12):1269-1277. doi:10.1007/s13258-018-0721-1

**43.** Nam DE, Yoo DH, Choi SS, Choi BO, Chung KW. Wide phenotypic spectrum in axonal Charcot-Marie-Tooth neuropathy type 2 patients with KIF5A mutations*. Genes Genomics*. 2018;40(1):77-84. doi:10.1007/s13258-017-0612-x

**44.** Lee AJ, Nam SH, Park JM, et al. Compound heterozygous mutations of SH3TC2 in Charcot-Marie-Tooth disease type 4C patients*. J Hum Genet*. 2019;64(9):961-965. doi:10.1038/s10038-019-0636-y

**45.** Lee SJ, Kanwal S, Yoo DH, Park HR, Choi BO, Chung KW. A POLG2 Homozygous Mutation in an Autosomal Recessive Epilepsy Family Without Ophthalmoplegia*. J Clin Neurol*. 2019;15(3):418-420. doi:10.3988/jcn.2019.15.3.418

**46.** Song GJ, Gupta DP, Rahman MH, et al. Loss-of-function of EBP50 is a new cause of hereditary peripheral neuropathy: EBP50 functions in peripheral nerve system*. Glia*. 2020;68(9):1794-1809. doi:10.1002/glia.23805

**47.** Lee AJ, Nam DE, Choi YJ, Nam SH, Choi BO, Chung KW. Alanyl-tRNA synthetase 1 (AARS1) gene mutation in a family with intermediate Charcot-Marie-Tooth neuropathy*. Genes Genomics*. 2020;42(6):663-672. doi:10.1007/s13258-020-00933-9

**48.** Kanwal S, Choi YJ, Lim SO, et al. Novel homozygous mutations in Pakistani families with Charcot-Marie-Tooth disease*. BMC Med Genomics*. 2021;14(1):174. doi:10.1186/s12920-021-01019-5

**49.** Kwon HM, Kim HS, Kim SB, et al. Clinical and Neuroimaging Features in Charcot-Marie-Tooth Patients with GNB4 Mutations*. Life (Basel)*. 2021;11(6) doi:10.3390/life11060494

**50.** Kim HS, Kim HJ, Nam SH, et al. Clinical and Neuroimaging Features in Charcot-Marie-Tooth Patients with GDAP1 Mutations*. J Clin Neurol*. 2021;17(1):52-62. doi:10.3988/jcn.2021.17.1.52

**51.** Kim HJ, Nam SH, Kwon HM, et al. Genetic and clinical spectrums in Korean Charcot-Marie-Tooth disease patients with myelin protein zero mutations*. Mol Genet Genomic Med*. 2021;9(6):e1678. doi:10.1002/mgg3.1678

**52.** Nam DE, Park JH, Park CE, et al. Variants of aminoacyl-tRNA synthetase genes in Charcot-Marie-Tooth disease: A Korean cohort study*. J Peripher Nerv Syst*. 2022;27(1):38-49. doi:10.1111/jns.12476

**53.** Jung NY, Kwon HM, Nam DE, et al. Peripheral Myelin Protein 22 Gene Mutations in Charcot-Marie-Tooth Disease Type 1E Patients*. Genes (Basel)*. 2022;13(7) doi:10.3390/genes13071219

**54.** Lim SO, Jung NY, Lee AJ, et al. Genetic and Clinical Studies of Peripheral Neuropathies with Three Small Heat Shock Protein Gene Variants in Korea*. Genes (Basel)*. 2022;13(3) doi:10.3390/genes13030462

**55.** Kwon HM, Park JH, Chung KW, Choi BO. Wide Phenotypic Spectrum of PNMHH Patients With p.R941L Mutation in MYH14*. J Clin Neurol*. 2022;18(2):238-240. doi:10.3988/jcn.2022.18.2.238

**56.** Kim HJ, Kim SB, Kim HS, et al. Phenotypic heterogeneity in patients with NEFL-related Charcot-Marie-Tooth disease*. Mol Genet Genomic Med*. 2022;10(2):e1870. doi:10.1002/mgg3.1870

**57.** Park J, Kim HS, Kwon HM, et al. Identification and clinical characterization of Charcot-Marie-Tooth disease type 1C patients with LITAF p.G112S mutation*. Genes Genomics*. 2022;44(8):1007-1016. doi:10.1007/s13258-022-01253-w
